# A nationwide survey of the association between nonalcoholic fatty liver disease and the incidence of asthma in Korean adults

**DOI:** 10.1371/journal.pone.0262715

**Published:** 2022-01-21

**Authors:** Jae-Hyung Roh, Hanbyul Lee, Bae Yun-Jeong, Chan Sun Park, Hyo-Jung Kim, Sun-Young Yoon

**Affiliations:** 1 Department of Cardiology in Internal Medicine, School of Medicine, Chungnam National University, Chungnam National University Sejong Hospital, Sejong, Korea; 2 Department of Statistics, Kyungpook National University, Daegu, Korea; 3 Health Innovation Bigdata Center, Asan Institute for Life Sciences, Asan Medical Center, Seoul, South Korea; 4 Department of Internal medicine, Inje University, Haeundae Paik Hospital, Busan, Korea; 5 Division of Pulmonology, Department of Internal Medicine, INJE Haeundae Paik Hospital, Busan, Korea; 6 Department of Allergy and Pulmonology in Internal Medicine, Chungnam National University, Chungnam National University Sejong Hospital, Sejong, Korea; Hualien Tzu Chi Hospital Buddhist Tzu Chi Medical Foundation, TAIWAN

## Abstract

**Background:**

Asthma and nonalcoholic fatty liver disease (NAFLD) are chronic diseases known to be associated with metabolic abnormalities. We aimed to clarify the association between NAFLD and asthma incidence in a large population-based cohort.

**Methods and findings:**

We selected 160,603 individuals without comorbidities from the National Health Insurance Service-National Sample cohort between 2009 and 2014. NAFLD was defined using a surrogate marker, fatty liver index (FLI). During a median of 5.08 years’ follow-up, 16,377 subjects (10.2%) were newly diagnosed with asthma and categorized into three groups according to FLI. The cumulative incidence of asthma was higher in subjects with higher vs. lower FLIs (FLI < 30, 10.1%; 30 ≤ FLI < 60, 10.8%; FLI ≥ 60, 10.5%). Higher FLI was associated with an increased incidence of asthma (Hazard ratios (HR)_highest vs. lowest FLI_, 1.25; 95% CI, 1.15–1.36). The results using another definition of NAFLD, as measured by the hepatic steatosis index (HSI), were similar to the primary results. This association was more pronounced in women than in men (HR 1.46; 95% CI, 1.13–1.64 vs. HR 1.07; 95% CI, 0.94–1.20).

**Conclusions:**

This study demonstrated that NAFLD, as measured by FLI and HSI, may influence the incidence rates of asthma in adults, especially in women.

## Introduction

Nonalcoholic fatty liver disease (NAFLD) is the most common chronic liver disease worldwide, and its prevalence in Asian-Pacific countries is estimated to be 20%–30% [1,2].

NAFLD is associated with an increase in multisystem diseases, including diabetes, ischemic cardiovascular disease, and chronic kidney disease [3,4]. Recently, an association between NAFLD and atrial fibrillation, a non-ischemic cardiac disease, has also been reported [5].

Asthma is the most common chronic airway disease, with approximately 350 million cases reported worldwide in 2015 [6]. Recent studies have reported the epidemiological link between metabolic syndrome (MetS) and asthma. Further, obesity is a well-known risk factor for asthma and may mediate its severity [7,8]. Several studies have reported the association of asthma with other components of MetS, such as dyslipidemia and insulin resistance, as well as obesity [9–11]. NAFLD and MetS also share many features, and there is growing evidence of a bidirectional relationship between the two diseases [12,13]. However, to date, no studies have investigated the direct association between NAFLD and the development of asthma.

We accordingly hypothesized that NAFLD was associated with asthma incidence. Since there was no previous study that showed an association between the two diseases, we tried to confirm the association between NAFLD and asthma while minimizing the influence of other factors between the two diseases. Therefore, we investigated the association between NAFLD and asthma incidence using a large, claim-based cohort consisting of Korean adults in whom the traditional risk factors and comorbidities for asthma and NAFLD, including smoking history, hypertension, diabetes, and cardio-cerebrovascular disease, were absent, as based on previous studies.

## Methods

### Database and study cohort

This study was approved by the Institutional Review Board of Chungnam National University Hospital, Sejong, Korea (protocol no. 2020-09-023) and was conducted in accordance with the Declaration of Helsinki guidelines. Written informed consent was waived by the ethics committee because all the analyses used anonymous data. We conducted a retrospective population-based cohort study using data from the National Health Insurance Service-National Sample Cohort 2.0 (NHIS-NSC 2.0) 2002–2015, as previously reported [14,15]. The NHIS-NCS is a large-scale, population-based cohort study that uses a systematic sampling method for random selection of representative databases constituted by approximately 1 million people from 2002 to 2015, which is 2.2% of the total Korean population [16].

This database contains data collected from cohort members on sociodemographic characteristics, all medical claims including diagnosis, medical treatment, and health care utilization, and is linked with data from the National Health Screening database. The database also includes mortality data such as the date and cause of death based on the death registration database of Statistics Korea.

### Study subjects

All subjects aged 20 years and older who underwent national health screening at least once between January 2009 and December 2014 were enrolled in this study. The first health check-up was considered as the index check-up, and the year of the index check-up was the index year. Subjects who met the following exclusion criteria were excluded from the analysis: i) diagnosed with asthma within two years before the index year; ii) diagnosed with comorbid conditions including other pulmonary disease, liver disease, heart failure, hypertension, diabetes, cerebrovascular disease, ischemic heart disease, valvular heart disease, or peripheral artery disease within two years before the index year; iii) had a prescription of oral hypoglycemic, antihypertensive, or lipid-lowering agents within two years before the index year; iv) history of smoking (current or former smoker); and v) had missing data in the index check-up and follow-up period.

National health screenings included physical examination, medical history, blood test, chest X-ray, and collection of data on health-related behaviors such as exercise habits, smoking, and alcohol consumption using self-administered questionnaires. Each diagnosis was defined using the 10^th^ revision of the International Classification of Diseases (ICD-10). Each diagnosis code used in the analysis is shown in S1 Table.

### Definition of adult-onset asthma cases and asthma-related healthcare use

Asthma was defined when both of the following conditions were met: i) individuals with at least two or more claims with asthma according to the ICD-10 code (J45.x–J46.x) for the primary or sub-diagnosis; ii) individuals who were prescribed at least one of the asthma-related medications: inhaled corticosteroids (ICS), ICS and LABA combined into a single inhaler (ICSs/LABAs), systemic LABAs, oral leukotriene antagonists (LTRA), short-acting β 2-agonists (SABAs), xanthine derivatives, and systemic corticosteroids [17]. In terms of asthma-related healthcare use, outpatient visits were defined as patients diagnosed with asthma-related ICD-10 codes (J45-46) and prescribed at least one of the asthma-related medications (see the above). Asthma-related hospitalization or emergency department (ED) visits were described under admission and ED visits according to the asthma-related ICD-10 codes (J45-46) either as primary or sub-diagnosis and accordingly, patients were prescribed oral or injected systemic corticosteroids to exclude healthcare use for other principal purposes [15,18].

### Data collection and measure

Body mass index (BMI) was calculated as weight (kg) divided by the square of height (m). Obesity was defined as a BMI ≥25 kg/m^2^ according to the World Health Organization criteria for Asian populations [19]. Alcohol consumption and daily activities were measured using self-reported questionnaires.

The fatty liver index (FLI), developed by Bedogni et al., is a reliable, noninvasive predictor of NAFLD. In previous studies, FLI demonstrated an accuracy of 0.84 (AUROC: Area under the receiver operator characteristic curve) in Western populations and an accuracy of 0.82–0.83 in Asians in terms of prediction of NAFLD [20–23]. FLI consists of BMI, waist circumference, triglyceride, and gamma-glutamyltransferase (GGT) and is calculated using the following formula:

$$FLI=\frac{e^{0.953\;In(TG)+0.139\times BMI+0.718\times In(GGT)+0.053\times WC-15.745}}{1+e^{0.9533In(TG)+0.139\times BMI+0.718\times In(GGT)+0.053\times WC-15.745}}*100$$

The FLI score ranges from 0 to 100, and the original study suggested an FLI score of ≥60 as the cut-off value to diagnose hepatic steatosis. In this study, subjects were then categorized into three groups: 0 ≤ FLI < 30, 30 ≤ FLI <60, and FLI ≥ 60 respectively, based on previous studies [22].

However, few studies have been conducted to validate this application of FLI in Asian populations, considering ethnic characteristics such as lower BMI and waist circumference compared to those in Western populations. Therefore, we conducted supplemental analysis using different FLI cut-off criteria: 0 ≤ FLI < 25, 25 ≤ FLI < 35, and FLI ≥ 35 for males and 0 ≤ FLI < 10, 10 ≤ FLI < 20, and FLI ≥ 20 for females, as proposed by Yang et al. in a study conducted among Taiwanese people [23]. The hepatic steatosis index (HSI), another surrogate marker of fatty liver, HSI was also calculated as a supplementary criterion. HSI consists of liver enzymes, such as alanine transaminase (ALT), aspartate aminotransferase (AST), and BMI, calculated using the following formula: HSI = 8x (ALT/ AST ratio) + BMI (+2, if female; +2, if diabetes mellitus). HI values of ≥ 36 rules in hepatic steatosis [24].

### Statistical analysis

Data are presented as mean ± standard deviation for continuous variables and as number (percentage) for categorical variables. One-way analysis of variance and chi-square tests were used to compare the differences between the FLI groups. The incidence rate of asthma was determined by dividing the total number of newly diagnosed asthma cases during 2009–2014 by the total population during the same period and expressed as cases per 100,000 person-years. Cumulative incidence rates were calculated and compared between quartile-based groups using Kaplan-Meier estimates and log-rank tests. Cox proportional hazards regression was used to estimate adjusted hazard ratios (HRs) and 95% confidence intervals (CIs) were used for asthma incidence. The covariates included in the model 1 were age and sex. Later, clinical characteristics of variables whose association with adult-onset asthma showed borderline statistical significance (P < .10), physical activity, alcohol consumption, systolic blood pressure, diastolic blood pressure, fasting blood glucose, and low-density lipoprotein cholesterol were incorporated into Model 1 to obtain Model 2. The multicollinearity of the variables included in the analysis was checked using VIF (variance inflation factor), and all of them were less than 10, indicating that they did not affect the results.

During the subgroup analyses, FLI was integrated into the statistical models as a continuous variable after log transformation. A two-sided P value of < 0.05, was considered to indicate statistical significance. Statistical analyses were performed using R software, version 3.3.3 (R Foundation for Statistical Computing, Vienna, Austria; www.r-project.org).

## Results

### Baseline characteristics

**Fig 1:**
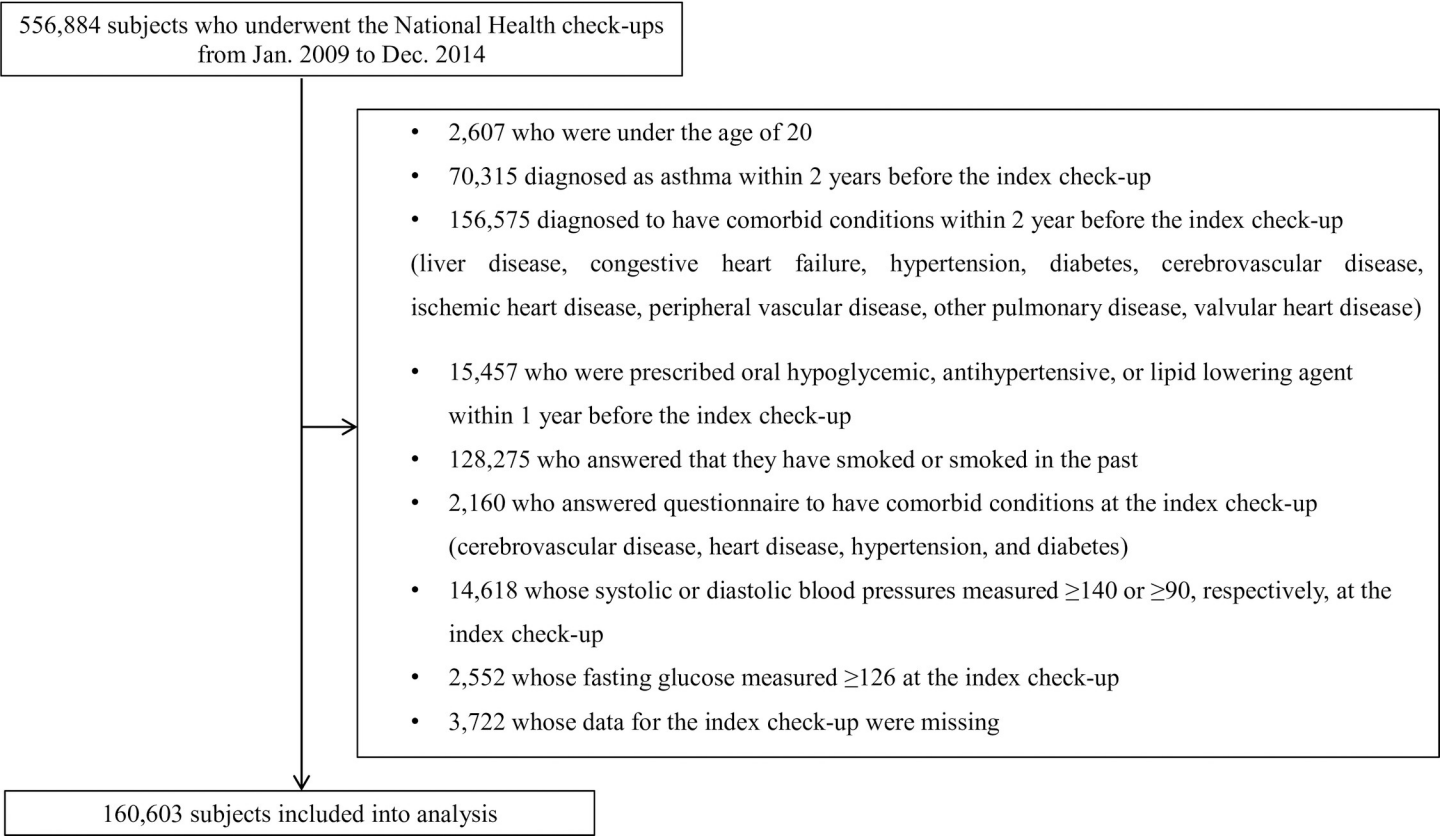
Overview of the study population.

Of the total 556,884 NHIS-NSC 2.0 sample cohort population, the data of 160,603 individuals were analyzed after excluding subjects who met the exclusion criteria (Fig 1). The study subjects were divided into three groups according to their FLI values (0 ≤ FLI <30, 30 ≤ FLI <60, and FLI ≥ 60). The detailed baseline characteristics of the FLI group are shown in Table 1. Groups with higher FLI had older individuals and a greater proportion of obese and male subjects than the groups with lower FLI. Mean blood pressure and the proportion of alcohol consumers also tended to increase with FLI. In terms of laboratory data, the lipid profile progressively worsened with increasing FLI values, and GGT and fasting glucose levels were also higher in patients with higher FLI.

**Table 1:** Baseline characteristics of study population.

Variables	Fatty liver index (FLI)			
	0≤ FLI <30 N = 136,094	30≤ FLI <60 N = 18,373	FLI ≥60 N = 6,136	P value
Age	40.7 ± 12.3	45.4 ± 12.3	42.6 ± 11.6	< 0.001
Sex (male, %)	2,574 (18.8)	9,462 (51.5)	4,062 (66.2)	< 0.001
BMI (Kg/m^2^)	21.9±2.5	26.1±2.4	28.8±3.5	< 0.001
WC (cm)	73.1 ± 7.1	85.6±5.6	92.5±7.2	< 0.001
SBP, mmHg	114.0±11.4	120.7±10.4	123.2±9.7	< 0.001
DBP, mmHg	71.1±8.1	75.3±7.4	77.2±6.9	< 0.001
Alcohol consumption (g/week)	26.6±65.8	48.1±105.6	76.5±149.6	< 0.001
Activity (met-min/week)	355.6±366.2	352.3±376.2	340.6±368.0	< 0.001
*Laboratory data*				
Fasting glucose, mg/dL	89.8±10.0	94.0±11.1	96.1±11.6	< 0.001
Total cholesterol, mg/dL	186.9±35.8	206.0±45.9	214.0±47.4	< 0.001
LDL cholesterol, mg/dL	113.6±168.5	125.5±97.5	123.9±80.7	< 0.001
HDL cholesterol, mg/dL	61.0±23.1	53.7±37.2	52.8+43.7	< 0.001
Triglyceride, mg/dL	85.8±43.7	164.9±90.8	238.2±193.3	< 0.001
GGT, U/L	17.8±11.3	38.4±33.1	71.4±72.4	< 0.001

**Abbreviations:** BMI = body mass index; DBP = diastolic blood pressure; FLI = fatty liver index; GGT = γ-glutamyltransferase; HDL = high-density lipoprotein; LDL = low-density lipoprotein; SBP = systolic blood pressure; WC = waist circumference.

### Relationship between FLI and the incidence of asthma

**Fig 2:**
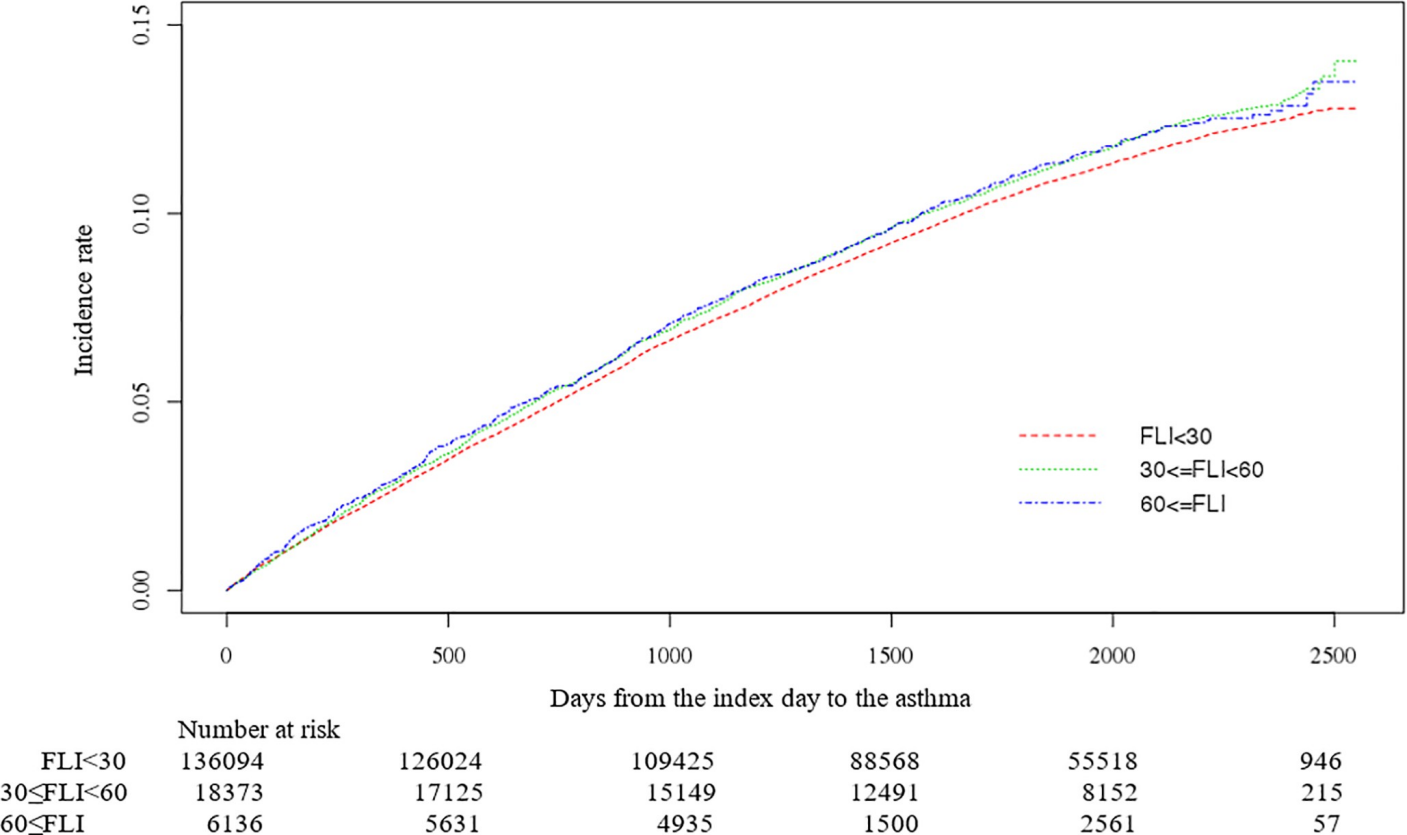
Cumulative incidence of adult-onset asthma by FLI group.

During the median follow-up, 5.08 (interquartile range, 3.08–6.16), a total of 16,377 subjects (10.2%) developed asthma. The incidence of asthma was high in groups with higher FLI (FLI <30 10.1% [13,744/136,094], 30≤ FLI <60 10.8% [1,986/18,373], FLI ≥60 10.5% [647/6,136]), and the cumulative incidence of asthma is shown in Fig 2.

Table 2 shows hazard ratios and their 95% CI from Cox proportional hazards regression analysis for subjects with adult-onset asthma according to the FLI group. After adjusting for age and sex (Model 1), the HR (95% CI) was 1.23 (1.13–1.33) for the highest group of FLI compared with that of the lowest. When the model was further adjusted for physical activity, alcohol drinking, systolic blood pressure, diastolic blood pressure, fast blood glucose, and low-density lipoprotein cholesterol (Model 2), the HR (95% CI) was 1.25 (1.15–1.36) for the highest group of FLI compared with that of the lowest. In addition to the FLI groups, the analysis conducted after categorizing the study subjects according to the different FLI cut-off criteria suggested in previous studies (HR 1.18, 95% CI 1.14–1.22) and HSI (HR 1.12, 95% CI 1.07–1.17), showed that asthma incidence tended to increase with an increase in each of these values (Table 2).

**Table 2:** Association between fatty liver index and incidence of adult-onset asthma.

			Univariate			Model 1*			Model 2^†^		
	Total (N)	Event (n, %)	HR	95% CI	*P value*	HR	(95% CI)	*P value*	HR	95% CI	*P value*
**FLI group**											
0≤ FLI <30	136,094	13,744(10.1)	Reference			Reference			Reference		
30≤ FLI < 60	18,373	1,986 (10.8)	1.04	0.99–1.09	0.06	1.13	1.07–1.18	<0.001	1.14	1.08–1.20	<0.001
FLI ≥60	6136	647 (10.5)	1.04	0.96–1.12	0.28	1.23	1.13–1.33	<0.001	1.25	1.15–1.36	<0.001
**FLI criteria** ^**‡**^											
low likelihood	103630	9846(9.5)	Reference			Reference			Reference		
intermediate likelihood	25797	2943(11.4)	1.11	1.06–1.14	< 0.001	1.09	1.05–1.13	0.001	1.10	1.06–1.14	< 0.001
High likelihood	31176	3588(11.5)	1.12	1.08–1.15	< 0.001	1.17	1.13–1.19	< 0.001	1.18	1.14–1.22	< 0.001
**HSI**											
HSI < 36	139608	14044(10.1)	Reference			Reference			Reference		
HSI ≥36	20995	2333(11.1)	1.10	1.06–1.15	< 0.001	1.09	1.04–1.14	<0.001	1.12	1.07–1.17	<0.001

*Cox proportional hazard models including age, and sex as covariates.

^†^Cox proportional hazard models including Model 1 plus activity, drinking, systolic blood pressure, diastolic blood pressure, fast blood glucose and low-density lipoprotein cholesterol as covariates.

^‡^Low likelihood: 0≤ FLI <25 for male, 0≤ FLI <10 for female; intermediate likelihood: 25≤ FLI< 35 for male, 10≤ FLI <20 for female; high likelihood: FLI ≥35 for male, FLI ≥20 for female.

**Abbreviations:** CI = confidence interval; FLI = fatty liver index; HR = hazard ratio; HSI = Hepatic steatosis.

We also conducted an additional analysis of FLI and asthma incidence according to the BMI group to confirm the possibility of obesity having an effect on the association between FLI and HSI and asthma incidence in this population. In all BMI groups, the incidence of asthma showed a tendency to increase with an increase in FLI, however, there was no statistical significance observed in the lowest BMI group (S2 Table).

Furthermore, we performed a subgroup analysis of the association between asthma incidence and FLI according to sex. As a result, the FLI was found to be associated with asthma incidence for both sexes, however, it was more pronounced in women (HR 1.46, 95% CI, 1.13–1.64 vs. HR 1.07, 95% CI, 0.94–1.20, Table 3).

**Table 3:** Sex specific associations between fatty liver index and incidence of asthma.

			Univariate			Model 1*			Model 2^†^		
	Total (N)	Event (n, %)	HR	95% CI	*P value*	HR	(95% CI)	*P value*	HR	95% CI	*P value*
**Female**											
0≤ FLI <30	110522	11794(10.7)	Reference			Reference			Reference		
30≤ FLI <60	8919	1175(13.2)	1.23	1.16–1.31	<0.001	1.17	1.10–1.24	<0.001	1.18	1.11–1.26	<0.001
FLI ≥60	2071	313(15.1)	1.49	1.33–1.66	<0.001	1.44	1.28–1.61	<0.001	1.46	1.13–1.64	<0.001
**Male**											
0≤ FLI <30	25572	1950(7.6)	Reference			Reference			Reference		
30≤ FLI <60	9454	811(8.6)	1.09	1.01–1.18	0.03	1.048	0.96–1.13	0.25	1.05	0.97–1.15	0.182
FLI ≥60	4065	334(8.2)	1.07	0.95–1.2	0.23	1.049	0.93–1.17	0.42	1.07	0.94–1.20	0.275

*Cox proportional hazard models including age as covariates.

^†^Cox proportional hazard models including Model 1 plus activity, BMI, drinking, systolic blood pressure, diastolic blood pressure, fast blood glucose and low-density lipoprotein cholesterol as covariates.

**Abbreviations:** CI = confidence interval; FLI = fatty liver index; HR = hazard ratio.

### Utilization of health care services

During the follow-up period, the highest FLI group had a higher asthma-related hospitalization rate than the other groups (0≤ FLI <30, 0.93%; 30≤ FLI <60, 1.06%; FLI ≥60, 1.9%, *P* <0.001). Moreover, the average frequency (1.04 vs. 1.64, *P* < 0.001), and days (12.7 vs. 17.8 per person; 8.38 vs. 13.56 per admission) were significantly higher in the group with high FLI (Table 4). Asthma-related intensive care unit transfer tended to increase with an increase in FLI (0.04%–0.08%, *P* <0.001), and there were significantly more outpatient visits in the higher FLI group (59.7% vs. 63.4%, *P* <0.001).

**Table 4:** Asthma-related healthcare use between FLI groups.

Variables	0≤ FLI <30	30≤ FLI <60	FLI ≥60	*P value*
N(%)*	13744(83.9)	1986(12.1)	647 (4.0)	
Hospitalizations				< 0.001
Never	13617(99.07%)	1965(98.94%)	635(98.1%)	
Ever^†^	127(0.93%)	21(1.06%)	12(1.9%)	
Mean ± SD^‡^	1.04 ± 1.01	1.3 ± 0.98	1.64 ± 1.15	< 0.001
Hospitalization days^§^				
per capita (Mean, SD)	12.7 ± 13.15	14.5 ± 12.24	17.8 ± 18.32	< 0.001
per each admission (Mean, SD)	8.38 ± 8.7	10.72 ± 8.65	13.56 ± 11.28	< 0.001
ICU hospitalizations				< 0.001
Never	13685(99.96%)	192(99.93%)	642(99.92%)	
Ever	59(0.04%)	14(0.07%)	5(0.08%)	
Mean ± SD	1.21 ± 0.51	1.22 ± 0.24	1.27 ± 0.71	0.283
ED visit^¶^				
Never	13744	1986	647	
Ever	-	-	-	
Mean ± SD	-	-	-	
No. of outpatient visits				< 0.001
Never	5523(40.3%)	750(37.9%)	242(36.6%)	
Ever	8211 (59.7%)	1231 (62.1%)	420 (63.4%)	
Mean ± SD	3.42 ± 4.87	3.45 ± 7.91	3.64 ± 8.2	0.068

*Total number of new onset asthma in each FLI group.

^†^Number of patients who underwent asthma-related hospitalization at least once.

^‡^Average number of admissions per person among patients who had asthma-related hospitalizations at least once.

^§^Average length of stay among patients who had asthma-related hospitalizations at least once (per person or per admission).

^¶^Included patients who underwent asthma-related ED visits without admission.

### Subgroup analysis of clinical variable affecting asthma incidence

The incidence of asthma in each clinical subgroup is shown in S1 Fig. In all subgroups except alcohol drinkers, asthma occurrence tended to increase as FLI increased.

S3 Table presents the HRs for asthma incidence according to each clinical variable. As the age and BMI of the study population increased, the HR tended to increase slightly, and the incidence of asthma was higher in women than in men (male, HR 0.69, 95% CI, 0.67–0.72). Total cholesterol and low-density lipoprotein cholesterol levels were also associated with increased HR. Daily physical activity has a protective effect on asthma incidence.

## Discussion

In this study, we investigated the association between NAFLD as measured using FLI, a validated surrogate marker, and the incidence of asthma in a large, representative sample of the Korean population. NAFLD was associated with an increase in the incidence of asthma, and in most clinical subgroups except alcohol drinkers, an increase in the incidence of asthma was observed with an increase in the FLI score. Furthermore, as the FLI score increased, the utilization of asthma-related health care services, such as hospitalization days and outpatient visits, also increased in these patients.

In the current study, the incidence of asthma was slightly higher in the middle group (30 ≤ FLI < 60) than in the highest FLI group. However, as a result of confirming the hazard ratios, it was found that asthma incidence increased as FLI increased, and the same result was obtained in the analysis using other types of FLI categories and HSI, another surrogate marker of NAFLD. Therefore, our findings support the possibility that NAFLD may influence the occurrence of asthma.

NAFLD is known to occur along the lines of MetS progression [25] and the association of NAFLD with extrahepatic complications such as cardiovascular disease, chronic kidney disease, and insulin resistance is well known [3,4,25]. Because potential associations with other diseases, including atrial fibrillation and periodontitis, have recently been suggested, there is increased interest in the effect of NAFLD on multiple organs [5,26].

Numerous studies have reported a correlation between MetS and impaired lung function. Of the MetS components, robust epidemiological data linking abdominal obesity with the incidence and exacerbation of asthma are available [27]. Other factors, such as dyslipidemia, hyperglycemia, and hypertension, have also been shown to be independently associated with asthma [9,28].

Given these results, a relationship between asthma and NAFLD may be suspected. Therefore, we evaluated the association between these two diseases using a large population after excluding known risk factors such as diabetes, hyperlipidemia, hypertension, and smoking exposure, and confirmed the association of NAFLD with asthma incidence. To the best of our knowledge, this is the first study to confirm the association between asthma incidence and NAFLD.

The association between NAFLD and asthma incidence identified in this study may be explained by several mechanisms underlying the two diseases. First, excessive circulatory TG and free fatty acids observed in NAFLD may have contribute to the systemic inflammation [29,30]. Increased proinflammatory cytokines and oxidative stress by excess free fatty acids may cause airway epithelial damage and airway inflammation, which may affect asthma development [31,32]. Second, insulin resistance, which plays a key role in the development of fatty liver disease, leads to excessive insulin secretion in the blood. Insulin is thought to contribute to airway hypersensitivity and remodeling by causing epithelial damage and smooth muscle proliferation in the airway [33].

Similar to the above-mentioned observations, our study also confirmed that an increase in age, weight, or poor lipid profile affects the incidence of asthma. Daily physical activity had a positive effect on asthma suppression.

Furthermore, we analyzed the correlation between FLI and asthma-related healthcare service utilization, and we found that the number of patients who experienced hospitalization was higher in the group with low FLI, but the number of hospitalizations per patient, number of days of hospitalization, and outpatient frequency increased with higher FLI. Increased health care use in the high FLI group suggests a possible correlation between FLI and asthma severity. However, since asthma severity was not reflected in this study, it is difficult to conclude the correlation between the two conditions. Further research is needed in the future.

Another important point in this study was that women showed a stronger correlation between FLI and asthma incidence than men. Several previous studies have reported a higher correlation between obesity and asthma in women [34,35], but others have provided conflicting results [36]. Some studies have reported a correlation between metabolic abnormality and the occurrence of asthma [9,37], but few studies have investigated gender differences. There are several possible mechanisms for the sex-specific differences in the association between asthma incidence and FLI. It is thought that the female sex hormone estrogen may be involved in Th2 inflammation and airway hypersensitivity [38]. It has also been suggested that the level of leptin, an adipokine with pro-inflammatory effects, is high in women, independent of body fat mass [39]. Overlapping genetic foci associated with asthma and obesity have been reported, and genetic differences are thought to have an effect [40,41], however, this remains unclear.

Currently, NAFLD is a disease of increasing global concern, and several guidelines recommend regular screening in patients with chronic conditions such as obesity, diabetes mellitus, and MetS [42]. In addition to weight reduction, oral hypoglycemic agents are currently being used to treat NAFLD [42]. Based on the results of the current study, improvement of NAFLD through such interventions is expected to have a positive effect on asthma, especially in female asthma patients. More studies are required in the future accordingly.

Several limitations of the current study need to be acknowledged. First, the incidence of asthma may be overestimated because asthma patients may be asymptomatic or have symptoms but do not need medical care for years. To minimize this error, all patients who were diagnosed with asthma or were prescribed asthma-related medication within two years before the index year were excluded based on previous studies [43]. However, the duration of two years may not have been sufficient. Second, in this study, determination of NAFLD status did not involve imaging tests such as abdominal ultrasonography or magnetic resonance imaging, which are currently the first-line diagnostic methods. However, the FLI used in the current study was validated as a predictor of NAFLD in several existing studies in both Asian and Western populations [20,22,23,44]. Third, in this study, we did not exclude heavy alcohol drinkers, so the effect of alcohol on fatty liver development could not be completely excluded. However, when analyzing the correlation between FLI and asthma incidence, alcohol drinking was corrected to minimize the effect of alcohol intake on the analysis results. Forth, due to the limitation of a retrospective study using big database, it is difficult to conclude that the association between the incidence of asthma and NAFLD observed in current study is direct, and the possibility of unknown factors affecting both conditions cannot be excluded. Finally, we were unable to include a family history of allergic disease, allergen exposure, and atopic status known as asthma risk factors in this study due to the nature of health insurance data. Finally, the generalizability of our findings is limited to the Korean population.

## Conclusion

In conclusion, the results of this study suggest that NAFLD, which was measured using the FLI, was associated with an increase in asthma incidence in a Korean population, especially in women. It was also demonstrated that progression of NAFLD may affect the healthcare utilization of asthma. These findings suggest that clinicians should be aware of the higher risk of asthma among patients with NAFLD. Further research on the impact of NAFLD management on the incidence and severity of asthma is thus, needed.

## Supporting information

S1 Fig Incidence of new onset asthma in various subgroups. (DOCX)

S1 Table Diagnoses defined by the 10th revision of the International Classification of Disease codes. (DOCX)

S2 Table Association between fatty liver index and incidence of adult-onset asthma according to BMI. (DOCX)

S3 Table Factors associated with the incidence of adult-onset asthma. (DOCX)
