# Supplementary material for: A nationwide survey of the association between nonalcoholic fatty liver disease and the incidence of asthma in Korean adults
Source: PLoS One. 2022 Jan 21;17(1):e0262715. doi: 10.1371/journal.pone.0262715 (PMC8782316; doi:10.1371/journal.pone.0262715)

**
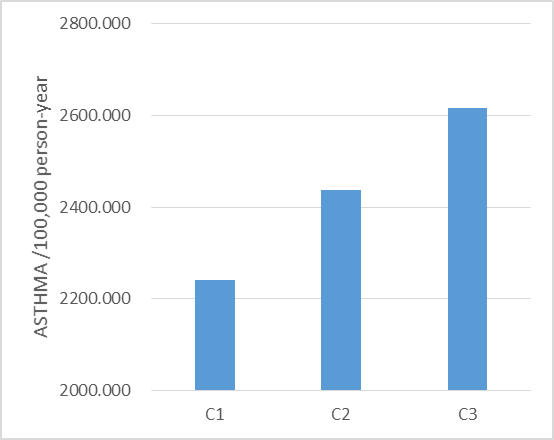

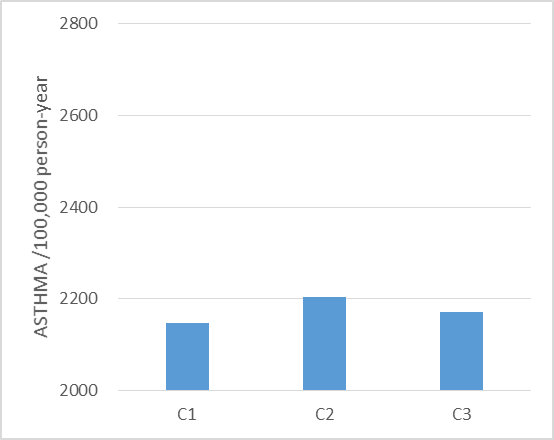

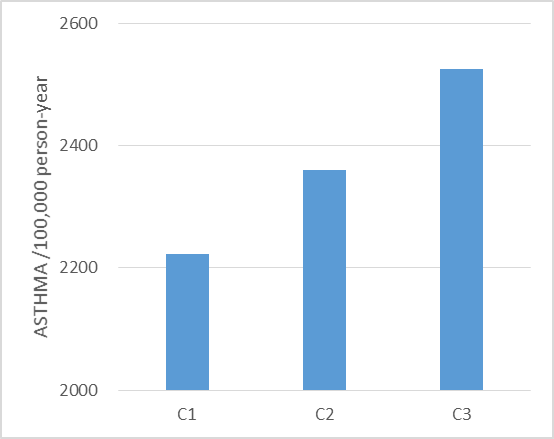

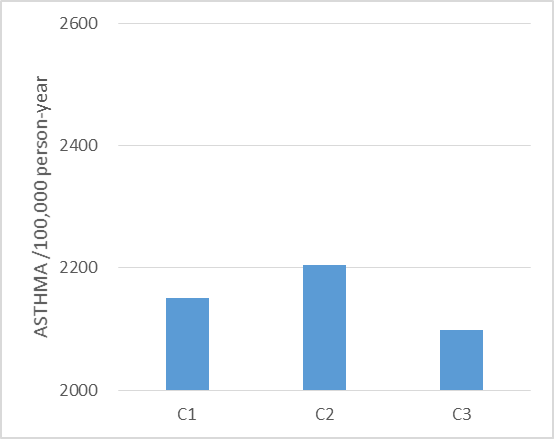

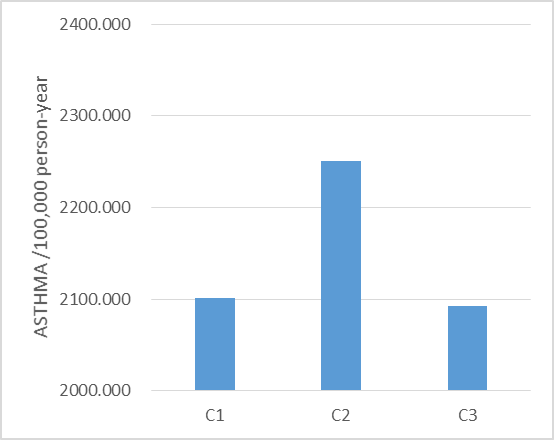

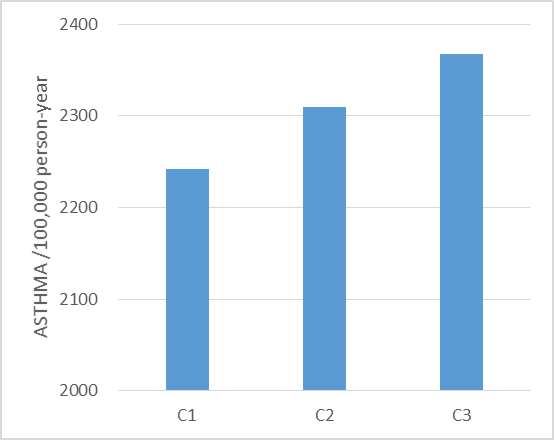

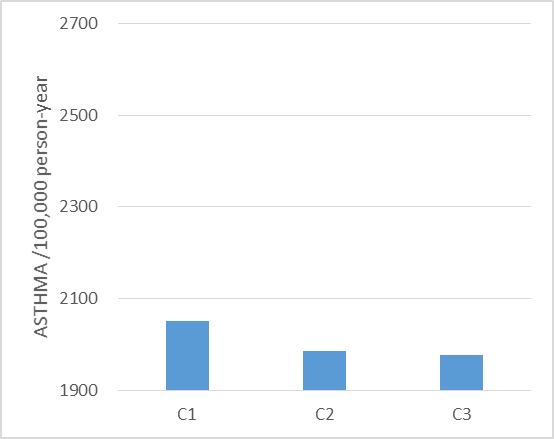

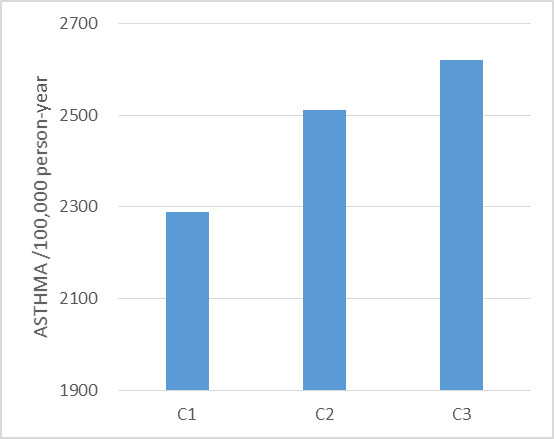

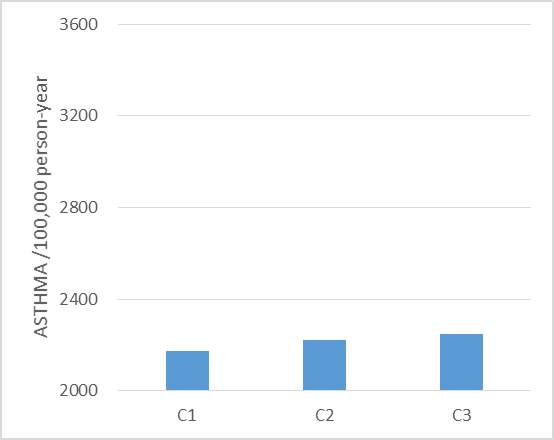

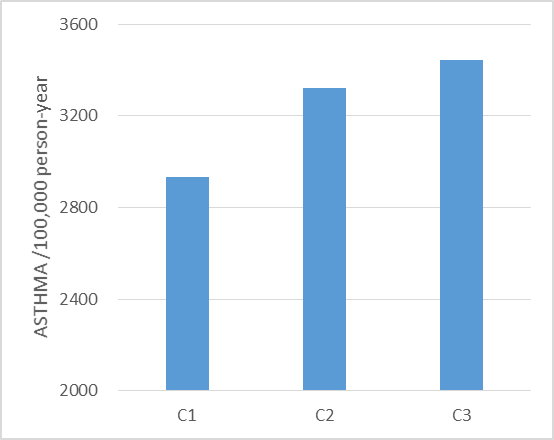

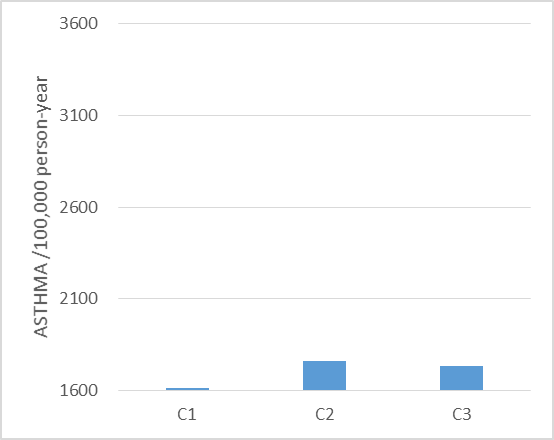

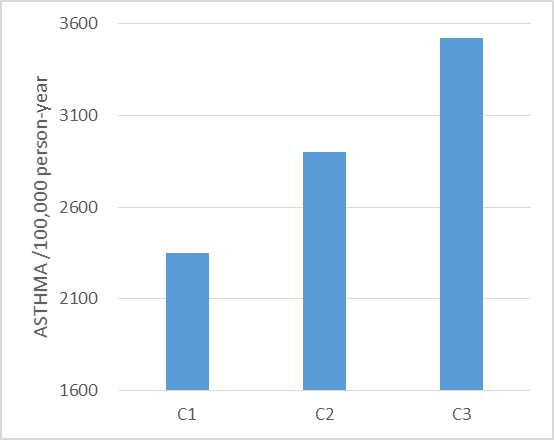
S1 Fig. Incidence of new onset asthma in various subgroups.**

**20 ≤ AGE < 65**

**AGE ≥ 65**

**Male**

**Female**

**Drinking (g/wk) = 0**

**Drinking (g/wk) > 0**

**Activity (met-min/wk) < 500**

**Activity (met-min/wk) ≥ 500**

**Systolic blood pressure, mmHg < 120**

**Systolic blood pressure, mmHg ≥ 120**

**Diastolic blood pressure, mmHg ≥ 80**

**Diastolic blood pressure, mmHg < 80**

**Fasting glucose, mg/dL < 100**

**Fasting glucose, mg/dL ≥ 100**


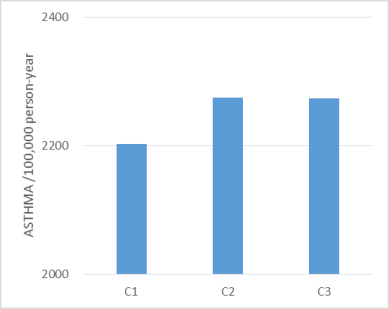

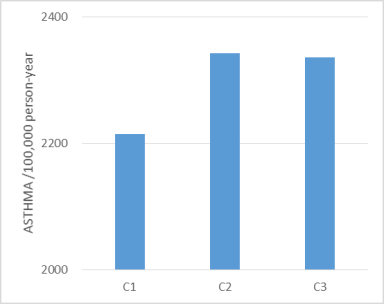

Supplement: S1 Fig — (DOCX) [file pone.0262715.s001.docx]
