# Supplementary material for: A nationwide survey of the association between nonalcoholic fatty liver disease and the incidence of asthma in Korean adults
Source: PLoS One. 2022 Jan 21;17(1):e0262715. doi: 10.1371/journal.pone.0262715 (PMC8782316; doi:10.1371/journal.pone.0262715)
Supplement: S1 Table — (DOCX) [file pone.0262715.s002.docx]

**S1 Table. Diagnoses defined by the 10th revision of the International Classification of Disease codes^.^**

| Diagnosis | International Classification of Disease, 10th revision code |
| --- | --- |
| Asthma | J45.x, J46.x |
| Cerebrovascular disease | G45.x, G46.x,, H34.0, I60.x-169.x |
| Congestive heart failure | I09.9, I11.0, I13.0, I13.2, I25.5, I42.0, I42.5-I42.9, I43.x, I50.x, P29.0 |
| Diabetes | E10.x-E14.x |
| Hypertension | I10.x-I15.x |
| Ischemic heart disease | I20.x-I25.x |
| Liver disease | B15.x-B19.x, K70.x-K77.x, K80.x-K87.x, I85.x, I86.4, I98.2, I98.3, Z94.4 |
| Myocardial infarction | I21.x-I23.x |
| Other pulmonary disease | I27.x, J40-J47, J60.x – J70.x |
| Peripheral vascular disease | I70.x, I71.x, I73.1, I73.8, I73.9, I77.1, I79.0, I79.2, K55.1. K55.8, K55.9, Z95.8, Z95.9 |
| Valvular heart disease | A52.0, I05.x-I08.x, I09.1, I09.8, I34.x-I39.x, Q23.0-Q23.3, Z95.2-Z95.4 |
