# Supplementary material for: A nationwide survey of the association between nonalcoholic fatty liver disease and the incidence of asthma in Korean adults
Source: PLoS One. 2022 Jan 21;17(1):e0262715. doi: 10.1371/journal.pone.0262715 (PMC8782316; doi:10.1371/journal.pone.0262715)
Supplement: S2 Table — (DOCX) [file pone.0262715.s003.docx]

**S2 Table. Association between fatty liver index and incidence of adult-onset asthma according to BMI.**

|  |  |  | **Univariate** | | | | **Model 1^*^** | | | **Model 2^†^** | | |
| --- | --- | --- | --- | --- | --- | --- | --- | --- | --- | --- | --- | --- |
|  | Total (N) | Event (n, %) | HR | 95% CI | | *P value* | HR | (95% CI) | *P value* | HR | 95% CI | *P value* |
| **BMI < 18.5** | | | | | | | | | | | | |
| 0 ≤ FLI < 30 | 10790 | 1004(9.3) | Reference |  |  | | Reference |  |  | Reference |  |  |
| 30 ≤ FLI < 60 | 14 | 2(14.3) | 1.358 | 0.339-5.438 | 0.665 | | 1.600 | 0.395-6.474 | 0.310 | 1.615 | 0.398-6.561 | 0.303 |
| FLI ≥ 60 | 1 | 0(0.0) | - | **-** | **-** | | **-** | **-** | **-** | **-** | **-** | **-** |
| **18.5 ≤ BMI < 23** | | | | | | | | | | | | |
| 0 ≤ FLI < 30 | 81060 | 8012(9.9) | Reference |  |  | | Reference |  |  | Reference |  |  |
| 30 ≤ FLI < 60 | 1351 | 124(9.2) | 0.886 | 0.742-1.058 | 0.280 | | 0.977 | 0.816-1.170 | 0.203 | 0.980 | 0.818-1.174 | 0.224 |
| FLI ≥ 60 | 97 | 10(10.3) | 1.003 | 0.556-1.920 | 0.12 | | 1.158 | 0.622-2.156 | < 0.001 | 1.169 | 0.627-2.179 | < 0.001 |
| **BMI ≥ 23** | | | | | | | | | | | | |
| 0 ≤ FLI < 30 | 44244 | 4728(10.7) | Reference |  |  | | Reference |  |  | Reference |  |  |
| 30 ≤ FLI < 60 | 17008 | 1860(10.9) | 1.011 | 0.958-1.066 | 0.703 | | 1.108 | 1.049-1.172 | <0.001 | 1.114 | 1.054-1.179 | <0.001 |
| FLI ≥ 60 | 6038 | 637(10.5) | 0.996 | 0.917-1.081 | 0.916 | | 1.214 | 1.115-1.323 | <0.001 | 1.230 | 1.127-1.342 | <0.001 |

^*^Cox proportional hazard models including age as covariates

^†^Cox proportional hazard models including Model 1 plus activity, BMI, drinking, systolic blood pressure, diastolic blood pressure, fast blood glucose and low density lipoprotein cholesterol as covariates

CI = confidence interval; FLI = fatty liver index; HR = hazard rati
