# Supplementary material for: A nationwide survey of the association between nonalcoholic fatty liver disease and the incidence of asthma in Korean adults
Source: PLoS One. 2022 Jan 21;17(1):e0262715. doi: 10.1371/journal.pone.0262715 (PMC8782316; doi:10.1371/journal.pone.0262715)
Supplement: S3 Table — (DOCX) [file pone.0262715.s004.docx]

**S3 Table. Factors associated with the incidence of adult-onset asthma.**

| Variables | Category | N | event  (n, %) | HR | 95% CI | P value |
| --- | --- | --- | --- | --- | --- | --- |
| Age | 20-34 | 52048 | 4664(9.0) | Reference |  |  |
|  | 35-49 | 67586 | 6486(9.6) | 1.030 | 0.992-1.069 | 0.127 |
|  | 50-64 | 34402 | 4271(12.4) | 1.290 | 1.238-1.345 | <0.001 |
|  | 65-74 | 5307 | 787(14.8) | 1.515 | 1.404-1.633 | <0.001 |
|  | ≥75 | 1260 | 169(13.4) | 1.509 | 1.294-1.759 | <0.001 |
| Sex | Female | 121515 | 13282(10.9) | Reference |  |  |
|  | Male | 39091 | 3095(7.9) | 0.697 | 0.671-0.725 | < 0.001 |
| BMI (Kg/m^2^) | <18.5 | 10805 | 1006(9.3) | 0.962 | 0.899-1.029 | 0.257 |
|  | 18.5-19.9 | 20759 | 2000(9.6) | 0.988 | 0.938-1.040 | 0.640 |
|  | 20-22.4 | 51205 | 5087(9.9) | Reference |  |  |
|  | 22.5-24.9 | 44237 | 4595(10.4) | 1.027 | 0.987-1.069 | 0.186 |
|  | ≥25 | 33597 | 3689(11.0) | 1.106 | 1.060-1.154 | <0.001 |
| WC (cm) | Continuous* | | | 1.004 | 1.002-1.006 | <0.001 |
| SBP, mmHg | <120 | 95384 | 9825(10.3) | Reference |  |  |
|  | 120-139 | 65219 | 6552(10.1) | 0.959 | 0.927-0.986 | 0.005 |
| DBP, mmHg | <80 | 117353 | 11996(10.2) | Reference |  |  |
|  | 80-89 | 43250 | 4381(10.1) | 0.967 | 0.934-1.001 | 0.055 |
| Alcohol consumption | 0 | 100871 | 10875(10.8) | Reference |  |  |
| (g/week) | ≤140 | 50916 | 4752(9.3) | 0.888 | 0.859-0.919 | <0.001 |
|  | >140 | 8816 | 750(8.5) | 0.795 | 0.738-0.856 | <0.001 |
| Activity (met-min/week) | <500 | 118158 | 1242(10.4) | Reference |  |  |
|  | 500-999 | 31815 | 3058(9.6) | 0.933 | 0.896-0.970 | <0.001 |
|  | ≥1000 | 10630 | 1077(10.1) | 0.966 | 0.908-1.028 | 0.277 |
| Fasting glucose, mg/dL | < 100 | 132874 | 13495(10.2) | Reference |  |  |
|  | 100-125 | 27729 | 2882(10.4) | 1.018 | 0.9578-1.060 | 0.384 |
| Total cholesterol, mg/dL | Continuous^*†^ | | | 1.252 | 1.152-1.362 | <0.001 |
| LDL cholesterol, mg/dL | Continuous^*†^ | | | 1.099 | 1.050-1.120 | <0.001 |
| HDL cholesterol, mg/dL | Continuous^*†^ | | | 0.961 | 0.905-1.019 | 0.184 |
| Triglyceride, mg/dL | Continuous^*†^ | | | 1.067 | 1.037-1.099 | <0.001 |
| GGT, U/L | Continuous^*†^ | | | 0.999 | 0.972-1.026 | 0.932 |

^*^Each clinical characteristics were incorporated in univariate Cox proportional hazard models as continuous variables

^†^Each clinical characteristics were incorporated in univariate Cox proportional hazard models after a log transformation.

BMI = body mass index; CI = confidence interval; DBP = diastolic blood pressure; FLI = fatty liver index; GGT = γ-glutamyltransferase; HDL = high-density lipoprotein; HR = hazard ratio; LDL = low-density lipoprotein; SBP = systolic blood pressure; WC = waist circumference.
